# Couple Communication in Cancer: Protocol for a Multi-Method Examination

**DOI:** 10.3389/fpsyg.2021.769407

**Published:** 2022-02-07

**Authors:** Shelby L. Langer, Joan M. Romano, Francis Keefe, Donald H. Baucom, Timothy Strauman, Karen L. Syrjala, Niall Bolger, John Burns, Jonathan B. Bricker, Michael Todd, Brian R. W. Baucom, Melanie S. Fischer, Neeta Ghosh, Julie Gralow, Veena Shankaran, S. Yousuf Zafar, Kelly Westbrook, Karena Leo, Katherine Ramos, Danielle M. Weber, Laura S. Porter

**Affiliations:** ^1^Center for Health Promotion and Disease Prevention, Edson College of Nursing and Health Innovation, Arizona State University, Phoenix, AZ, United States; ^2^Department of Psychiatry and Behavioral Sciences, University of Washington School of Medicine, Seattle, WA, United States; ^3^Department of Psychiatry and Behavioral Sciences, Duke University School of Medicine, Durham, NC, United States; ^4^Department of Psychology and Neuroscience, University of North Carolina at Chapel Hill, Chapel Hill, NC, United States; ^5^Department of Psychology and Neuroscience, Duke University, Durham, NC, United States; ^6^Clinical Research Division, Fred Hutchinson Cancer Research Center, Seattle, WA, United States; ^7^Department of Psychology, Columbia University, New York, NY, United States; ^8^Department of Psychiatry and Behavioral Sciences, Rush University Medical Center, Chicago, IL, United States; ^9^Public Health Sciences Division, Fred Hutchinson Cancer Research Center, Seattle, WA, United States; ^10^Department of Psychology, University of Washington, Seattle, WA, United States; ^11^Edson College of Nursing and Health Innovation, Arizona State University, Phoenix, AZ, United States; ^12^Department of Psychology, University of Utah, Salt Lake City, UT, United States; ^13^Institute of Medical Psychology, University Hospital Heidelberg, Heidelberg University, Heidelberg, Germany; ^14^Division of Medical Oncology, University of Washington School of Medicine, Seattle, WA, United States; ^15^Duke Cancer Institute, Duke University Medical Center, Durham, NC, United States

**Keywords:** cancer, couples, adjustment, cognitive processing, intimacy, distress, relationship adjustment

## Abstract

Cancer and its treatment pose challenges that affect not only patients but also their significant others, including intimate partners. Accumulating evidence suggests that couples’ ability to communicate effectively plays a major role in the psychological adjustment of both individuals and the quality of their relationship. Two key conceptual models have been proposed to account for how couple communication impacts psychological and relationship adjustment: the social-cognitive processing (SCP) model and the relationship intimacy (RI) model. These models posit different mechanisms and outcomes, and thus have different implications for intervention. The purpose of this project is to test and compare the utility of these models using comprehensive and methodologically rigorous methods. Aims are: (1) to examine the overall fit of the SCP and RI models in explaining patient and partner psychological and relationship adjustment as they occur on a day-to-day basis and over the course of 1 year; (2) to examine the fit of the models for different subgroups (males vs. females, and patients vs. partners); and (3) to examine the utility of various methods of assessing communication by examining the degree to which baseline indices from different measurement strategies predict self-reported adjustment at 1-year follow up. The study employs a longitudinal, multi-method approach to examining communication processes including: standard self-report questionnaires assessing process and outcome variables collected quarterly over the course of 1 year; smartphone-based ecological momentary assessments to sample participant reports in real time; and laboratory-based couple conversations from which we derive observational measures of communicative behavior and affective expression, as well as vocal indices of emotional arousal. Participants are patients with stage II-IV breast, colon, rectal, or lung cancer and their spouses/partners, recruited from two NCI-designated comprehensive cancer centers. Results will be published in scientific journals, presented at scientific conferences, and conveyed to a larger audience through infographics and social media outlets. Findings will inform theory, measurement, and the design and implementation of efficacious interventions aimed at optimizing both patient and partner well-being.

## Introduction

Patients with cancer often report disease- and treatment-related side effects including fatigue, pain, and cognitive impairment (Bower, 2008; IOM, 2008). Emotional distress is also common, manifesting as depression, anxiety, and fears of disease progression and death (Syrjala and Yi, 2014). For the many patients who are married or in committed relationships, cancer affects the partner as well. Indeed, partners are known to experience an array of difficulties, including anxiety, depression, fatigue, sleep disturbance, and employment disruption (Girgis et al., 2013; DeMoor et al., 2017; Geng et al., 2018; Areia et al., 2019). Partners’ relationship satisfaction may also wane over time following the patient’s cancer treatment (Langer et al., 2010).

Accumulating evidence indicates that couples’ ability to communicate effectively plays a major role in the adjustment of both patients *and* partners to the illness experience (Baucom et al., 2012). Specifically, communication behaviors that are associated with better adjustment include open discussion of cancer-related concerns (often referred to as disclosure), and the ability to listen and respond supportively to one’s partner. Maladaptive communication behaviors include holding back from disclosure, and avoiding or responding negatively to one’s partner’s disclosure. A variety of self-report measures have been utilized to assess adaptive and maladaptive communication behavior, including those assessing *disclosure* and *holding back* from disclosure; *protective buffering* which is hiding concerns and/or negative emotions from one’s partner (Hagedoorn et al., 2000); and *social constraints* which are perceptions that the partner’s responses to one’s own disclosures are avoidant, discouraging or disapproving (Lepore and Revenson, 2007). In general, individuals who hold back from expressing cancer-related concerns to their partner, or perceive their partner as non-responsive, avoidant, or critical of their expressions have poorer individual and relationship functioning (Porter et al., 2005; Hinnen et al., 2009; Langer et al., 2009; Traa et al., 2015).

Two key conceptual models have been proposed to account for how communication difficulties may lead to poorer outcomes. According to the social-cognitive processing (SCP) model, lack of cognitive processing is the primary mechanism linking communication difficulties to psychological distress. Individuals who perceive their partner as unreceptive to discussing cancer-related concerns talk less about them with their partner, reducing cognitive processing needed to assimilate and accommodate the cancer experience into their world view, leading to increased psychological distress (Lepore, 2001). The proposed mechanism, lack of cognitive processing, is operationalized as experiencing unwanted intrusive thoughts about cancer and/or avoidance of reminders of cancer. In contrast, the relationship intimacy (RI) model (Manne and Badr, 2008) posits that decreased intimacy (the experience of closeness and caring) is the primary mechanism by which poor communication leads to both psychological *and* relationship distress.

To date, most studies citing these models as a guiding framework have been cross-sectional in design, have involved breast or prostate cancer samples (with some exceptions, e.g., Manne and Badr, 2010), and have tested individual pathways vs. the models as a whole. For instance, a review and meta-analysis found moderately strong associations between social constraints and intrusive thoughts, and social constraints and psychological distress (Adams et al., 2015). However, there is little evidence that cognitive processing is the mechanism (e.g., mediator) by which social constraints affect adjustment. Support for the RI model is perhaps stronger. Links between maladaptive communication and lower intimacy have been established (Perndorfer et al., 2019), as have links between maladaptive communication and psychological and relationship distress (Kayser et al., 1999; Manne et al., 1999, 2010, 2014; Hagedoorn et al., 2000; Porter et al., 2005; Langer et al., 2009; Traa et al., 2015). And some studies *have* demonstrated mediating paths, for example, that mutual avoidance is associated with greater psychological distress through decreased intimacy (Manne et al., 2010) or that disclosure is associated with lower psychological distress through increased intimacy (Manne and Badr, 2010), though these studies were cross-sectional and therefore unable to provide definitive evidence regarding directionality.

Another critique of the extant literature in this area is that most studies have employed standard global questionnaire-based measures of communication. It is widely acknowledged that relationships and communication processes are dynamic and can change over time and with circumstances (Lepore and Revenson, 2007; Badr et al., 2013), thus an understanding of these processes requires going beyond the single-time retrospective measures that are commonly used. Ecologically valid approaches offer a number of advantages. We focus here on ecological momentary assessment (EMA). First, EMA minimizes recall biases inherent in global, retrospective measures which require participants not just to remember but also to summarize their behavior. When considering such questions, individuals use a variety of heuristics to estimate their answers, leading to systematic biases influenced, for example, by current mood (Shiffman et al., 2008). Second, compared to both global self-report and laboratory-based assessments, EMA increases ecological validity as participants are reporting in their real-world environment. Third, the longitudinal nature of EMA data can be used to examine temporal sequences of behaviors and experiences (Shiffman et al., 2008). To our knowledge, a handful of studies has employed daily diary approaches to assess constructs relevant to either the RI model (Belcher et al., 2011; Perndorfer et al., 2019) or the SCP model (Pasipanodya et al., 2012; Badr et al., 2013), but they all involved breast cancer samples and were relatively small in size, ranging from 45 dyads (Belcher et al., 2011) to 69 dyads (Perndorfer et al., 2019). Because the majority of breast cancer cases are female, sole focus on this cancer site confounds gender and patient role. In addition, these studies employed electronic diaries but were internet-based vs. smartphone-delivered. Given the ubiquity of cell phones (94%) and in particular smartphones (81%) in the United States (Pew Research Center, 2019), the ease and immediacy of gathering EMA on a device already at hand may facilitate completion and minimize participant burden. This is true for not just younger adults but also persons aged 50 and higher, with smartphone ownership among the latter having increased from 53% in 2015 to 67% in 2018 (Pew Research Center, 2019).

Direct observation of interactions between patients with cancer and their partners provides another valuable source of information with some unique advantages. Observational assessment provides a relatively objective measure of behaviors of interest without being filtered through the report of either the patient or partner, and thus avoids threats to validity, such as social desirability biases, that may be associated with the use of self-report measures. Direct observation also provides real-time assessment and thus can obviate inaccuracies due to recall over time. In addition, many self-report measures of couples’ communication were not developed for use in couples coping with cancer, and so may not capture the types of problems or interactions that may be most meaningful to these couples, whereas direct observation can provide stimulus prompts that focus on those issues. Finally, direct observation methods have been used for decades in studying couples’ interactions, and have shown good reliability and validity in capturing important dimensions of couples’ communication associated with adjustment (Snyder et al., 2005).

Another promising objective approach to studying couples’ communication is the assessment of expressed emotional arousal during couples’ interactions *via* measurement of a vocal feature, fundamental frequency (f_0_). Similar to facial expressions, features of the voice contain significant information about internal emotional experiences (Juslin and Scherer, 2005). f_0_ is the lowest frequency harmonic of the speech sound wave, is associated with perceived pitch (Atkinson, 1978), and is a valid indicator of overall emotional arousal rather than specific emotion (Banse and Scherer, 1996; Juslin and Scherer, 2005; Baucom, 2010). It is also associated with psychophysiological measures of arousal including heart rate, blood pressure, and cortisol (Weusthoff et al., 2013). To date, there has been only one study that has assessed expressed emotional arousal in the context of couples’ cancer-related conversations. Fischer et al. (2015) examined associations between f_0_ and social support behaviors in the context of conversations between women with breast cancer and their male partners. Findings indicated that: (a) women with a higher f_0_ overall displayed more behaviors likely to elicit positive support (such as stating their needs clearly), and (b) women displayed fewer adaptive support-eliciting behaviors when their partners exhibited higher overall f_0_. These findings suggest that it may be adaptive for female patients to experience and express emotional arousal during conversations of cancer-related concerns, but that high emotional arousal on the part of their partners may interfere with this process. The findings, while preliminary, suggest that this is a promising approach with the potential to inform measurement and theory of the processes through which communication affects adaptation to cancer. In addition to examining overall levels of emotional arousal, this approach lends itself to a more detailed examination of how arousal evolves across the interaction, providing further insights into communication-related processes.

The purpose of the present project is to provide a comprehensive and methodologically rigorous evaluation of both the SCP and RI models, including delineation of mediators (how they work) and moderators (for whom they work). Specific aims are: (1) To examine the overall fit of the SCP and RI models in explaining patient and partner psychological and relationship adjustment as they occur on a day-to-day basis and over the course of 1 year. (2) To examine the fit of the models for different subgroups: males vs. females, and patients vs. partners. (3) To examine the utility of the various methods of assessing communication (global self-report vs. EMA vs. objective measures) by examining the degree to which each of these baseline indices predicts self-reported psychological and relationship adjustment at 1-year follow up.

We hypothesize that the SCP model will predict individual psychological adjustment *via* actor effects, and that the RI model will predict individual psychological adjustment *via* actor effects and relationship adjustment *via* both actor and partner effects (aim 1). It is also likely that the strength of these associations will vary between individuals or subgroups (aim 2). While there are numerous variables that could potentially moderate these mediating effects, based on the literature and potential clinical relevance, we chose to examine gender and role (patient vs. spouse). With regard to gender, stronger positive associations between perceived social constraints and distress have been found among male vs. female cancer patients (Zakowski et al., 2003). Further, females report discussing their cancer with a wider circle of confidantes than do males (Harrison et al., 1995) which could result in increased cognitive processing of their cancer experience outside the relationship. Thus, lack of cognitive processing may be a more important driver of distress for males vs. females. With regard to role, patients and partners often have different assumptions about how they should support each other, with some partners believing that it is harmful for the patient to discuss their cancer or any negative aspects of the situation (Peters-Golden, 1982); this is reflected in findings that partners report higher levels of protective buffering than do patients (Langer et al., 2009). Thus, it is possible that the negative effects of such buffering may be experienced more by patients than by partners, but this requires further examination. Regarding aim 3, because EMA and objective measures are less subject to reporting biases (e.g., social desirability and recall), we hypothesize that they will better predict outcomes.

This study is the first comprehensive multi-method longitudinal study of couples’ communication in the context of cancer. The design incorporates key recommendations in the literature (Lepore and Revenson, 2007; Badr et al., 2013): (a) a dyadic approach that includes both partners and treats the couple as the unit of analysis; (b) the inclusion of dyads dealing with different types of cancer, including three cancers that affect both male and female patients so that role can be disentangled from gender; (c) the use of observational methods that enable analysis of interactional patterns and expressed emotional arousal as well as comparisons between these measures and self-report in predicting outcomes; (d) the use of EMAs that can capture the interactional nature of communication processes occurring day-to-day, and the temporal sequencing of communication behaviors, mediating variables and adjustment; and (e) assessment of communication using self-report questionnaires collected four times over the course of 1 year, affording analysis of change in communication patterns and mediators across time.

## Methods and Analysis

### Design and Participants

The study design is longitudinal, with repeated questionnaire-based assessments at baseline and 4, 8, and 12 months post-baseline to capture quarterly reports across a 1-year follow-up period without undue participant burden. Patients are recruited from the Duke Cancer Institute in Durham, NC and the Seattle Cancer Care Alliance in Seattle, WA, two NCI-designated comprehensive cancer care centers. Inclusion criteria for patients are:

• age 18 or older
• stage II-IV breast, colon, rectal, or lung cancer
• currently receiving or having received a form of systemic therapy (or hormone therapy for breast cancer) within 2 years of diagnosis of current stage
• life expectancy of at least 6 months per primary oncologist
• ability to speak and comprehend English
• being married or in a committed, cohabiting relationship with a same- or opposite-sex partner

We chose to focus on breast, colorectal, and lung cancers because they are relatively common solid tumor cancers and because, with the exception of breast cancer, occur frequently in both males and females.

Inclusion criteria for partners are:

• age 18 or older
• ability to speak and comprehend English
• being married to or in a committed, cohabiting relationship with the patient

Exclusion criteria are provider non-approval (patients), cognitive impairment prohibiting completion of study assessments (patients and partners), and logistical constraints preventing participation (patients and partners).

Patients identified as meeting initial medical inclusion criteria per medical records are sent a study brochure and letter signed by their primary oncologist introducing the study and informing them that they will be contacted by a research team member by phone (with opt-out instructions in case they do not want to be contacted). Those contacted and deemed fully eligible based on further screening confirmation during the recruitment phone call (e.g., partner status) are provided a detailed description of the study aims, procedures, risks and benefits, and probed for understanding. If the patient decides that they would like to participate, the research team member obtains permission to speak with the partner. If permission is granted, the research team member describes the study aims, procedures, risks and benefits to the partner. If a given patient declines, their partner is not contacted. If both dyad members agree to participate, the in-person baseline assessment visit is scheduled. This visit commences with a formal face-to-face consent process (a repeat of details conveyed previously plus additional probing for understanding and discussion) and signing of consent documents.

### Timeline and Procedures

**FIGURE 1:**
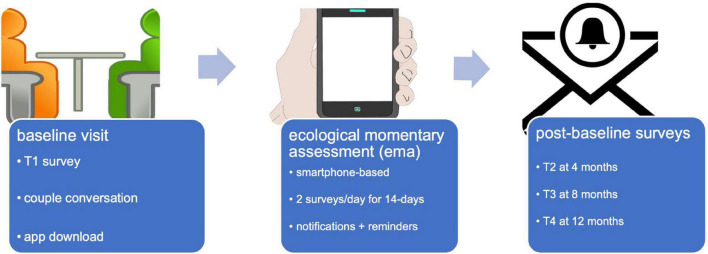
Study activities. Icons made by Freepik from www.flaticon.com.

Study participation lasts for 12 months and is depicted in Figure 1. In-person baseline assessment includes: (1) completion of the first set of questionnaires, (2) an audio- and video-recorded couple conversation, and (3) downloading and instruction in the use of a study-specific smartphone app for EMA, to commence that same day. The EMA activity includes twice-daily push notifications and lasts for 14 days, a duration designed to capture sufficient data to meet study aims while minimizing participant burden. Follow-up includes self-report questionnaire assessments only, completed online *via* REDCap at 4, 8, and 12 months post-baseline. Each activity is described in turn below. Participation is estimated at approximately 8 h total: a 2-h baseline assessment visit, twice-daily EMA surveys 5–10 min each for 14 days, and 3 follow-up questionnaires (30–45 min each).

### Medical Record Extraction

Data are extracted from medical record summary, oncology, and clinic visit notes to obtain screening and follow-up information: cancer site, current cancer stage, confirmation of current cancer stage within 2 years, current or previous therapies (systemic and/or hormone) and surgeries, primary oncologist, gender, primary language, year of birth, and marital/partner status.

### Questionnaires

Table 1 lists study questionnaires. In general, these measures were chosen based on their strong psychometric properties and their utility in previous investigations. Several are very commonly used in studies examining psychosocial functioning in the context of cancer. These are noted with an asterisk in Table 1.

**TABLE 1:** Questionnaire-based assessments administered *via* REDCap: Key constructs, internal consistency, and administration time point.

Questionnaire administered *via* REDCap	# Items	Alpha	Time
Demographic characteristics	8	–	T1
Utilization of psychological services	6	–	T1–T4
Charlson Comorbidity Index (Charlson et al., 1987), *13 comorbid conditions*	13	–	T1
Emotional Expressivity Scale (Kring et al., 1994), *dispositional emotional expressivity*	17	0.90–0.93	T1
**Communication**			
Protective Buffering (Suls et al., 1997), *enacted and received buffering*	14	0.80–0.87 (Langer et al., 2009)	T1–T4
Emotional Disclosure Scale (Pistrang and Barker, 1992), *disclosure and holding back re 10 cancer-related concerns*	20	0.85–0.91 (Porter et al., 2005)	T1–T4
*Social Constraints Scale (Lepore and Ituarte, 1999), *perceived constraints*	15	0.88–0.92	T1–T4
Communication Patterns Questionnaire (Christensen, 1987), *demand-withdraw and constructive communication*	35	0.81–0.84 (Heavey et al., 1996)	T1–T4
**Cognitive processing**			
*Impact of Events Scale (Horowitz et al., 1979), *intrusion and avoidance*	15	0.82–0.86 (Sundin and Horowitz, 2002)	T1–T4
**Intimacy**			
Miller Social Intimacy Scale (Miller and Lefcourt, 1982), *intimacy*	17	0.86–0.91	T1–T4
**Psychological and relationship adjustment**			
*Profile of Mood States, 2nd edition (POMS-2; Heuchert and McNair, 2012), *total mood disturbance*	35	0.76–0.95	T1–T4
*Dyadic Adjustment Scale (Spanier, 1976), *relationship adjustment*	32	0.96	T1–T4
Center for Epidemiologic Studies Depression-10 (Andresen et al., 1994), *depressive symptomatology*	10	0.86–0.91 (Miller et al., 2008)	T1–T4
**Well-being**			
*Functional Assessment of Cancer Therapy, general population (Cella et al., 1993), *physical, social, emotional, and functional well-being*	21	0.69–0.82	T4
**Supplemental measures**			
Stanford Brief Activity Survey (Taylor-Piliae et al., 2006), *usual physical activity*	2		T1
Functional Assessment of Chronic Illness Therapy—COST (de Souza et al., 2014), *financial distress*	11	0.90	T1–T4
Parenting Concerns Questionnaire (Muriel et al., 2012), *parenting concerns*	10	0.79	T1–T4
Revised Adult Attachment Scale (Collins and Read, 1990; Collins, 1996), *anxious and avoidant attachment*	18	0.78–0.85	T1–T4
Coronavirus Impact Scale (Hamilton et al., 2011), *COVID impacts*	12	Unknown	T1–T4

T1 = baseline; T2–T4 = 4, 8, and 12 months post-baseline, respectively. References for alpha values in column 3 are from the developer (cited in column 1) unless otherwise specified. *Commonly used in studies examining psychosocial functioning in the context of cancer.

All questionnaires are administered using Research Electronic Data Capture (REDCap), a secure web-based tracking and on-line data acquisition system (Harris et al., 2009). Our project-specific application resides on a secure Arizona State University server with password-protected access for pertinent personnel across study sites. Immediately following consent, participants are assigned unique numerical study identifiers comprising a unique dyad-identifier and a numeric code for role (patient vs. partner), which are then linked to the REDCap system. Patients and partners complete the first set of questionnaires during the in-person visit on REDCap using a tablet computer. An experimenter provides face-to-face instruction on use of the system and answers any questions as they arise, setting the stage for remote on-line completion thereafter. Participants are instructed to complete questionnaires independently, without conferring with their partner. Unique links are shared *via* email for all follow-up questionnaire administrations.

Supplemental measures added after the commencement of enrollment and designed to answer ancillary questions include the Revised Adult Attachment Scale (Collins and Read, 1990; Collins, 1996), the Parenting Concerns Questionnaire (Muriel et al., 2012), the Stanford Brief Activity Survey (Taylor-Piliae et al., 2006), the Functional Assessment of Chronic Illness Therapy—COST (de Souza et al., 2014), and the Coronavirus Impact Scale (Stoddard et al., 2021) from the PhenX Toolkit (Hamilton et al., 2011).

### Ecological Momentary Assessment

We developed a study-specific smartphone application using the LifeData platform. The app is free to use and compatible with both iOS and Android devices. Participants are asked to download the app during the baseline laboratory session, with guidance from an experimenter. They are also given a user instruction and FAQ document to take home and refer to as needed; study contact information is included for assistance.

Participants use their own smartphones to complete the EMA unless they either do not own one or own a device with a different operating system. Those without an appropriate smartphone borrow a study iPod Touch device for this activity. The app interface across these systems is comparable.

EMA is to commence following the laboratory-based visit. Participants receive a push notification to complete EMA twice daily over the course of 14 days: once at 12:00 p.m. and once at 8:00 pm, both times within a 2-h active window. Push notifications begin immediately after app download, so depending upon the time of day of the baseline assessment, participants begin receiving notifications either that afternoon or that evening. This timing, frequency, and duration was based on pilot work conducted with a non-medical sample (Langer et al., 2020). If participants do not begin to complete the assessment within the 2-h response window, the notification expires. Reminder notifications are included for each assessment; these arrive every 20 min, up to 5 times, until a participant responds or the notification expires.

At each EMA, patients and partners are asked to answer a series of questions. Branching logic minimizes participant burden. First, participants are asked if they talked to their partner during a given time frame (since awakening for the 12:00 p.m. time point or since the last assessment for the 8:00 p.m. time point). If no, a single item designed to assess reasons for not talking is posed (I didn’t have any contact with my partner; I had nothing to talk about; I didn’t feel well; I didn’t want to bring up topics that could be upsetting; other). If yes, questions designed to assess perceptions of the conversation follow. These are listed in Table 2.

**TABLE 2:** Smartphone-delivered ecological momentary assessment (EMA) items.

Item	Construct
To what extent was this conversation related to your/your partner’s cancer?	Cancer relatedness
How important was this conversation to you?	Importance
**Predictors**	
To what extent did you express your feelings?	Enacted disclosure
To what extend did you hold back from expressing your feelings?	Enacted holding back
To what extent did you act more positive than you felt?	Protective buffering
To what extent did you avoid talking about the issue?	
To what extent did you withhold potentially upsetting information from your partner?	
To what extent did you hide your worries?	
To what extent did you hide your anger?	
To what extent did you support your partner?	Supported partner
To what extent did you criticize your partner?	Criticized partner
To what extent did you understand your partner?	Understood partner
To what extent did you feel that your partner avoided talking about the issue?	Social constraints
To what extent did you feel that your partner supported you?	Perceived partner support
To what extent did you feel that your partner criticized you?	Perceived partner criticism
To what extent did you feel that your partner understood you?	Perceived partner understanding
**Mediators**	
How close do you feel to your partner right now?	Intimacy
How connected do you feel to your partner right now?	
How often did you have distressing thoughts about the cancer?	Intrusive thoughts
How often did you think about the cancer when you didn’t mean to?	
How often did you try to push away or avoid thoughts about the cancer?	Avoidance
How often did you avoid letting yourself get upset when you thought about the cancer or were reminded of it?	
**Outcomes**	
Dyadic Adjustment Scale item #31, posed only at the evening assessment • All things considered, what was your degree of happiness with your relationship today? (extremely unhappy, fairly unhappy, a little unhappy, happy, very happy, extremely happy, or perfectly happy; coded 0–6)	Relationship satisfaction
Profile of Mood States (Cranford et al., 2006)	Distress
• How vigorous do you feel right now?	
• How anxious do you feel right now?	
• How worn out do you feel right now?	
• How angry do you feel right now?	
• How sad do you feel right now?	
• How cheerful do you feel right now?	
• How fatigued do you feel right now?	
• How on edge do you feel right now?	
• How annoyed do you feel right now?	
• How discouraged do you feel right now?	
• How lively do you feel right now?	
• How uneasy do you feel right now?	
• How hopeless do you feel right now?	
• How resentful do you feel right now?	
• How exhausted do you feel right now?	
**Other/Ancillary**	
Please rate your current level of physical pain (no pain to pain as bad as you can imagine, 0–10 scale)	Pain
Please rate the overall quality of your sleep last night (not all restful to extremely restful, 0–4 scale; afternoon assessment only)	Sleep quality
To what extent has your physical health limited your usual activities today?	Physical health limitations

*All items posed on a 1–5 scale unless otherwise noted.*

### Laboratory-Based Couple Conversation

Couples are asked to participate in a 15-min cancer-related conversation that is video-recorded for later coding. Separate audio-recordings afford analysis of vocally encoded emotional arousal as described below. To assist participants in selecting topics of discussion for these conversations, we provide a list of cancer-related issues known to be relevant based on past research (Pistrang and Barker, 1992; Andrykowski et al., 1999). These topics are listed in Table 3. Both members of the dyad are instructed to independently review the list and select three topics that are meaningful to them, either because they have not yet discussed the issue but consider it important, or because it is something that remains unresolved or merits further elaboration. Space is included for participants to indicate unique topics of interest that are not on the list. After topics are chosen, couples review their six independently selected topics together and agree upon a single final topic for the conversation.

**TABLE 3:** Possible discussion topics.

Your reaction to the diagnosis
Disruptions to your life caused by the cancer diagnosis and treatment
Managing cancer treatments
Managing treatment side effects
Dealing with medical staff
Having to give up or cut back from work or other important activities
Communicating with friends or family members about the cancer
Talking with children about the cancer
Maintaining a sex life
Being hospitalized
Plans for the future
Financial concerns
Getting support from friends and family
Completing household tasks
Completing daily activities
Dealing with changes in your/your partner’s physical appearance
Fears or worries about disease progression or death
Concerns about the quality of medical care
Concerns about your partner’s response to the illness

Couples are asked to sit in chairs placed approximately two feet apart and facing one another at a slight angle. They are instructed to speak to one another vs. the camera, and to allow the conversation to flow as it normally would outside of the research setting.

Prior to picking the topic and the conversation, couples engage in a modified DiapixUK task (Baker and Hazan, 2011) designed to elicit spontaneous speech of each partner for 1 min. These recordings are used to obtain an f_0_ baseline for analyses of vocally encoded emotional arousal. In this task, one dyad member is handed a picture and asked to describe what is depicted in it (for example, a beach scene) to their partner. After 1 min, the experimenter hands three pictures to the partner and asks them to select the one that was described. Roles are then reversed with a different set of pictures.

### Observational Coding

Two independent yet complementary coding systems are used to characterize participants’ communicative behaviors and affective expressions captured during the 15-min couple conversations: (1) the Asymmetric Behavior Coding System (Leo et al., 2020) and (2) the Relational Affective Topography System (Leo et al., 2020). Drawing from the Valence Affective Connection model (Leo et al., 2019), the systems delineate communicative behavior and affect as positive or negative and as promoting togetherness or engagement with the partner vs. individuation or separation from the partner.

Coding is being conducted in two waves with separate coding teams for the two systems, each consisting of 4–6 trained raters. For both systems, ratings are made independently by each coder and for each 3-min segment of the 15-min conversation. Separate passes are made for patients and partners. Order of viewing is randomized across coders, with approximately 30% of cases rated by all coding team members to assess inter-rater reliability. Coding teams meet weekly by videoconference to address problematic (highly discrepant) codes or cases.

The Asymmetric Behavior Coding System (ABCS) was adapted for this project to measure communicative behavior in the context of conversations regarding cancer. The ABCS consists of 24 behavior codes that load onto four higher-level factors as shown in the upper half of Table 4. Coders rate each behavior on a scale of 1 (no behavior present) to 7 (high levels of behavior present). Codes are not mutually exclusive. The frequency of particular behaviors, their intensity, and the context in which they occur are all used to determine the appropriate rating.

**TABLE 4:** Key behaviors and affective expressions derived from the ABCS and RATS.

Asymmetric Behavior Coding System (ABCS)	
Positive approach/joining	Abbreviated coding descriptions/examples
• Maintaining/deepening	“Tell me more”
• Disclosure	“This is what I am thinking/feeling”
• Validation	“I hear you and I understand”
• Repair	“I’m sorry for what I just said/did a second ago”
• Collaboration	“Here’s what we can do to fix/solve x”
• Intimacy building	“I want to be closer/more connected to you”
• Justification	“Here’s what’s going on with me/why I did what I did”
• Requests	“Here’s what I want/need”
**Positive avoidance/individuating**	
• Accommodation	“Never mind, we don’t have to talk about it”
• Tough love	Holding partner accountable for actions without being judgmental
• Reassurance	“We’ve got this because we can handle it”
• Minimization	“We’ve got this because it’s not a big deal”
**Negative approach/joining**	
• Defensiveness	“It wasn’t my fault”
• Emotional protests	Whining
• Blame	“It’s your fault”
• Pressures for change	“You need to do this”
• Domineering	“This is what you think/feel”
• Belligerence	Taunting
• Contempt	“I don’t value you”
**Negative avoidance/individuating**	
• Controlling the conversation	“I’m talking right now”
• Withdrawal	“I don’t want to be here”
• Avoidance	“I don’t want to talk about this”
• Stonewalling	The kind of things you do on a plane/train/bus when you don’t want to talk to someone
• Submit	“Fine, whatever you say as long as we can be done talking about it”
**RELATIONAL AFFECTIVE TOPOGRAPHY SYSTEM (RATS)**	
Flat emotion	
• Boredom	
• Indifference	
Positive joining emotion	
• Warmth	
• Appreciation	
• Kindness	
Positive individuating emotion	
• Happiness	
• Enthusiasm	
• Amusement	
• Satisfaction	
Hard negative emotion	
• Anger	
• Disgust	
• Frustration	
• Outrage	
Soft negative emotion	
• Sadness	
• Fearfulness	
• Loneliness	
• Guilt	
• Vulnerability	

While the ABCS focuses on the content and function of specific communicative behaviors, the Relational Affective Topography System (RATS) focuses on the affective quality of expressions. The RATS involves three steps. First, coders record whether positive emotions, negative emotions, and flat emotions as defined in the lower half of Table 4 are observed in a given 3-min segment (yes or no for each). Second, coders classify any positive emotions observed as positive joining (making emotional room for the other partner) or positive individuating (taking emotional space in the conversation), and any negative emotions observed as hard (conveying a sense of injustice or ineffectiveness) or soft (conveying unguardedness and inviting a protective response). Note that for both of these distinctions, the two options are not mutually exclusive for a segment, i.e., both can be observed within a given segment. Third, if flat emotions (characterized by low activation and arousal) were observed in step 1, the extent to which specific constituent emotions, boredom and indifference, were observed are rated on a 0–7 (none to high levels of the emotion) scale. If positive joining emotions were observed in step 1, the extent to which warmth, appreciation, and kindness were observed are rated on the same 0–7 scale, and so on for positive individuating, negative emotion, and soft negative emotion. Please see the lower half of Table 4 for a list of these specific constituent emotions.

### Fundamental Frequency

We derive fundamental frequency (f_0_) mean from the couple conversations as a marker of expressed emotional arousal during the interaction for each dyad member. Audio recordings are made using Lavalier microphones (Shure BLX88 and Tascam DR-40 Linear PCM recorder), with each microphone targeting one dyad member and recording onto a separate channel, resulting in relative differences in volume depending on who is speaking at a given time. We then extract f_0_ as a continuous measure across the 15-min conversation in estimated values (hertz, Hz) for every quarter-second using Praat (Boersma and Weenink, 2019), with a bandpass filter of 75–300 Hz to restrict values to the normal range of adult speech (Owren and Bachorowski, 2007; Weusthoff et al., 2013). We also extract intensity in decibel (dB) for every quarter-second to identify patient and partner speech (procedures similar to Bryan et al., 2018). Speaker changes (marking talk turns) are identified based on zero-crossover points in intensity values between the two channels and a minimum threshold of intensity differences observed *via* visual inspection of the intensity plots. Instances of overlapping speech or external background noises result in subthreshold difference scores and are removed during this procedure. Where speaker diarization is not possible using this procedure, speaker tracks are manually segmented using Audacity (Team, 2018). f_0_ mean values are then aggregated at each talk turn (time when one person speaks before the other partner begins speaking); plots are visually inspected for outliers, and observations are removed as needed (e.g., not previously identified background noises or overlaps in speech, non-speech vocalizations). All data management procedures are conducted using Stata 16 (StataCorp, 2021).

### Incentives

Participants can earn up to $200 per person or $400 per couple for completing all parts of the study. Table 5 displays payment amounts per study activity.

**TABLE 5:** Incentives as a function of study activity.

Activity	Payment per individual	Payment per dyad
Laboratory visit	$50	$100
Ecological momentary assessment	$75 if completed at least 85% of assessments, otherwise $3 per assessment	$150
Follow-up questionnaires	$75 ($25 per assessment × 3)	$150
Total	$200	$400

### COVID Considerations

The first participants were enrolled in May of 2017. When in-person activities with human subjects were restricted due to the SARS-CoV-2 pandemic in March of 2020, we moved to IRB-approved remote enrollment. This allowed us to continue to carry out all study activities with the exception of the in-person couple conversation. The consent process was conducted *via* telephone and participants signed the consent form electronically through REDCap.

### Data Analysis

#### Sample Size and Power

We project a complete-case sample of approximately 264 dyads for the questionnaire portion of the study, in other words, 264 dyads for which *both* members complete T1, T2, T3, *and* T4 questionnaires. This is based on anticipated numbers of patients meeting medical recruitment criteria at each site as well as estimates regarding: the proportion of patients married or in committed cohabiting partnerships (80%), the proportion of patient-partner dyads agreeing to participate (50%), disease-specific survival rates, and drop-out over time (30%). Based on power calculations for indirect effects (Vittinghoff and Neilands, 2015) conducted in NCSS/PASS version 2021, this sample size (264 retained from 434 enrolled) should afford power of 0.80 to detect indirect (mediated) associations of communication measures (Xs) with outcomes (Ys; relationship satisfaction and psychological distress) *via* measures of cognitive processing and intimacy (Ms) comprising small-to-moderate constituent (i.e., X→M and M→Y) associations of *r* ≥ 0.25, assuming moderate confounding of X→M and M→Y associations (*R*^2^ = 0.15).

### Overview

Associations hypothesized under the SCP and RI models will be examined with structural equation models (SEMs) separately for questionnaire data from T1-T4 and data from the EMA phase, primarily in a longitudinal actor-partner interdependence model (APIM) framework, with patients and partners treated as distinguishable dyad members (Ledermann et al., 2011). To examine longitudinal within-couple associations in the questionnaire data, random intercept cross-lagged panel models (Hamaker et al., 2015) will be estimated. EMA data will be analyzed using multilevel SEMs with repeated observations of patient- and partner-reported measures treated as being nested within dyads. Differences in hypothesized associations based on gender and role (patient vs. partner) will be examined using a variety of dyadic and non-dyadic analytic approaches as detailed below. Examination of how different assessments of communication (questionnaire, EMA, and objective assessment) prospectively predict psychological and relationship adjustment at follow-up will also be estimated using SEMs in an APIM framework.

All SEMs will be estimated in Mplus 8.7. Bootstrap standard errors for direct and indirect effects will be estimated, except for multilevel SEMs, where a Monte Carlo simulation approach will be used (see, e.g., Zyphur et al., 2016). For models that prove to be unestimable under maximum likelihood (ML) estimation (e.g., due to computational demands of numerical integration), Bayseian estimation will be used. Models fit using ML will be evaluated using comparative fit index (CFI), root mean squared error of approximation (RMSEA), and standardized root mean square residual (SRMR) values, with CFI > 0.95, RMSEA ≤ 0.06 (with upper confidence limit ≤ 0.08), and SRMR < 0.08 as benchmarks for acceptable model fit (Hu and Bentler, 1999). For models fit using the Bayes estimator, Bayesian versions of CFI and RMSEA values (Asparouhov and Muthen, 2021) will be examined.

#### Preliminary Analyses

Prior to conducting the primary analyses, we will use univariate and bivariate statistics and plots to examine distributions of, and associations among, key study variables. The reliability of each multi-item composite measure in the questionnaire data will be evaluated using Cronbach’s alpha, and reliability of composite measures from the EMA data will be evaluated using methods described by Cranford et al. (2006).

#### Examining Social-Cognitive Processing and Relationship Intimacy Models

##### Questionnaire Data

To examine the overall fit of the SCP model and the RI model in explaining patient and partner psychological and relationship adjustment over the course of 1 year using T1-T4 questionnaire data, we will use structural equation models (SEMs) within an APIM framework, in which measures from both dyad members (i.e., patient and partner) are included in each analysis. In these SEMs, a proposed latent factor with 6 indicators (scale scores for the questionnaire measures of holding back, disclosure, perceived constraints, enacted and received protective buffering, and constructive communication) reflecting overall communication will prospectively predict either a cognitive processing (avoidance and intrusive thoughts) composite (under the SCP model) or intimacy (under the RI model), which will, in turn, prospectively predict measures of psychological distress (POMS Total Mood Disturbance [TMD] or CESD score) and relationship adjustment (DAS total score). Prior to estimating the full SEMs for the questionnaire data, a measurement model for the proposed communication factor will be developed and refined using a confirmatory factor analysis approach. The degree of measurement invariance (e.g., configural vs. metric vs. scalar) for this proposed latent factor between patients and partners (and between men and women) will be evaluated in a sequential fashion. Non-invariance at each step in the sequence will be assessed using likelihood ratio tests (with α = 0.05) and changes in CFI (target ≤ 0.01). Potential sources of non-invariance (e.g., unequal loadings) will be investigated and the measurement model will be modified accordingly (e.g., dropping non-invariant indicators).

In line with the general APIM framework, both prospective actor (within-person, cross-construct) effects (e.g., *patient* communication predicting *patient* intimacy) and prospective partner (between-person, cross-construct) effects (e.g., *patient* communication predicting *partner* intimacy) will be estimated along with autoregressive (prospective within-person, within-construct) associations. Mediators and outcomes will be treated as manifest variables in these models. Latent person-level intercept factors will be included to account for random between-person variability in mean levels of model constructs. Within-couple indirect associations of communication and outcomes (psychological distress or relationship adjustment) *via* mediators (e.g., actor-actor effects and partner-actor effects) will be estimated using a product-of-coefficients approach (MacKinnon et al., 2007) and evaluated using 95% confidence intervals.

##### Ecological Momentary Assessment Data

**FIGURE 2:**
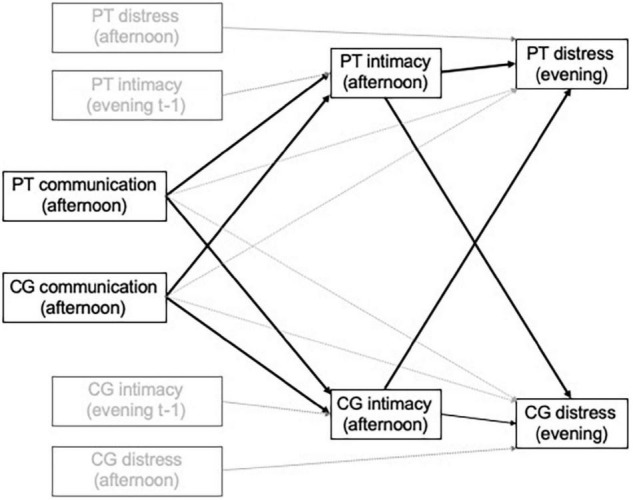
Illustration of analytic model to examine mediating dyadic processes using ecological momentary assessment data. PT, patient; CG, caregiver. Note. Variables and paths of conceptual interest are in black; control variables and paths less central to the conceptual model are in gray.

The EMA dataset will be structured with study days (level 1 units) nested within couples (level 2 units). Each study day observation will have values for patient and partner reports of afternoon communication (holding back, disclosure, protective buffering, support, perceived partner holding back, perceived partner support) as exogenous predictors, afternoon reports of cognitive processing (avoidance, intrusive thoughts) or intimacy as mediators, and evening reports of psychological distress (POMS TMD composite) or relationship satisfaction as outcomes, all from the same day’s afternoon and evening assessments. In addition, autoregressive associations are included such that afternoon reports of mediators are predicted from the preceding evening’s reports of the same variables (e.g., patient evening report of intimacy on day t-1 predicts patient afternoon report of intimacy on day t) and each evening outcome variable is predicted by the immediately preceding observation on the same variable. See Figure 2 for an example treating intimacy as the mediator and distress as the criterion, with variables and paths of conceptual interest in black, and control variables and paths less central to the conceptual models in gray. Indirect associations of afternoon communication with change in same-day evening outcomes (distress or relationship satisfaction) *via* mediators assessed on that same afternoon will be estimated using a product-of-coefficients approach and evaluated using 95% confidence intervals.

#### Differential Functioning of Social-Cognitive Processing and Relationship Intimacy Models by Gender and Role

To examine potential differential functioning of the SCP and RI models across gender (male vs. female) and role (patient vs. partner), we will employ two different modeling approaches within an APIM framework. For gender, we will add gender × communication and gender × mediator (e.g., gender × intrusive thoughts, gender × intimacy) terms to the standard “main effects” APIM models described above. For example, we would examine patient gender × patient communication, partner gender × partner communication, patient gender × patient intimacy, and partner gender × partner intimacy terms in a single model. To examine differential functioning by role, we will examine how individual cross-role equality constraints for parallel APIM actor and partner effects (e.g., patient communication-patient intimacy and partner communication-partner intimacy) affect model fit and test between-role differences in indirect effects. These models will be supplemented by exploratory analyses examining purely within-person (i.e., non-dyadic) models to examine indirect effects and model fit in gender and role group subsamples.

#### Utility of Different Communication Assessment Methods in Long-Term Prediction of Adjustment

Finally, to examine how communication measured early in the study predicts questionnaire measures of psychological and relationship adjustment at follow-up, we will estimate separate SEMs (again in an APIM framework), each of which uses a measure derived from each of the three different assessment methods (baseline questionnaire, EMA, and objective assessment) used in the study. First, we will estimate dyadic SEMs with actor and partner direct effects of early communication on each measure of adjustment at 12-month follow-up, adjusting for the person’s baseline assessment on the corresponding adjustment construct. In models based on questionnaire-measured communication, the general communication latent variable described above will be used. Objectively assessed communication, treated as a manifest variable, will be used in a parallel fashion in dyadic SEMs. Models based on EMA-measured communication will be estimated using two-level dyadic SEMs, with (the between-dyad variability in) repeated assessments of each patient’s and partner’s communication measure predicting psychological or relationship adjustment at follow-up, adjusting for the person’s baseline level on the outcome variable.

Analyses examining how f_0_ relates to psychological and relationship adjustment will include examination of how the trajectory of change in f_0_ over the course of the 15-min conversations predicts changes in psychological and relationship adjustment across the 1-year follow-up. First, trajectories of f_0_ across the conversation will be estimated for each partner by estimating a three-level (f_0_ for each talk turn nested within person nested within couple) multilevel growth curve model where f_0_ is regressed onto time (within conversation) and time^2^. Estimates will be generated for each person’s f_0_ intercept (i.e., initial f_0_ at the start of the conversation) and effect of time and time^2^ (linear and quadratic change, respectively, in f_0_ within the conversation). These values will be decomposed into within- and between-couple components. STATA will be used to analyze the primary models of interest in which longitudinal psychological and relationship adjustment separately are regressed on gender (as a covariate), role (patient vs. partner), f_0_ parameters within- and between-couple, wave (from baseline to 12 month), and their interactions. Model influence diagnostics and sensitivity analyses (including additional covariates) will be conducted to ensure stability of model results.

#### Supplemental Analyses

Analytic plans for the supplemental measures (bottom portion of Table 1) are described in turn below.

##### Stanford Brief Activity Survey

Descriptive statistics will be used to characterize patients and partners with regard to physical activity, an important health behavior known to ameliorate difficult effects of cancer treatment such as fatigue (Alfano et al., 2007).

##### Parenting Concerns Questionnaire

Data derived from this measure (posed only to participants who report having minor children in the home) will be used to examine associations between patient and partner parenting concerns and measures of communication, psychological distress, and relationship adjustment. This will build on findings from prior studies indicating that, among patients, parenting concerns are associated with higher levels of psychological distress (Park et al., 2016).

##### Functional Assessment of Chronic Illness Therapy—COST

This measure will be used to characterize the sample with regard to financial distress, both overall and as a function of sociodemographic and medical variables (cancer type and stage). We will also examine concordance or lack thereof in financial distress within couples and the trajectory of financial distress over time, from baseline to 1-year follow-up.

##### Coronavirus Impact Scale

This measure contains items designed to assess the extent to which the pandemic has caused change in multiple life domains: routines, family income/employment, food access, medical health care access, mental health treatment access, and access to extended family and non-family social supports. Additional items assess personal and family diagnoses of COVID, and perceived severity of pandemic-related stress and stress and discord in the family (none, mild, moderate, or severe). We will utilize descriptive statistics to characterize the sample with regard to COVID diagnoses and impacts on the life domains listed above. We will also conduct APIM analyses to examine concurrent intra- and inter-personal associations between pandemic-related stress and self-reported measures of communication (such as holding back) and well-being as measured by the FACT-GP.

##### Adult Attachment Scale

From the Revised Adult Attachment Scale, we will derive subscales of anxious and avoidant attachment styles for each participant. For analyses involving this measure, we will apply an Actor Partner Interdependence Mediation Model to APIM couples’ self-reported attachment, communication (disclosure and holding back), and physical well-being as measured by the FACT-GP. Indirect associations between a dyad member’s attachment (either anxious or avoidant) and their partner’s physical well-being are proposed as mediated by the dyad member’s own communicative behavior. We will examine model fit as described in the analytic overview above. Direct and indirect associations will be evaluated using 95% bootstrap confidence intervals.

## Discussion

To our knowledge, this is the first multi-method longitudinal study of couples’ communication in the context of cancer. Results will provide evidence to enable testing models of how couples’ communication is associated with adjustment to the cancer experience. Our long-term goal is to use these results to design and refine interventions that will improve couples’ communication and relationship functioning, alleviate cancer-related distress, reduce caregiver burden, and optimize patient and partner recovery from the rigors of cancer. Empirical support for the SCP model would indicate that interventions should focus on strategies that enhance cognitive processing. While these could include couple-based interventions that facilitate couples’ discussion of cancer-related issues, they may also include individual interventions that provide the opportunity for cognitive processing through disclosure to supportive others (e.g., a therapist or support group) or in non-social forms such as expressive writing. In contrast, support for the RI model would indicate that couple-based interventions are needed and should focus on enhancing intimacy through strategies to increase partners’ feelings of closeness and caring (e.g., increasing physical intimacy; engaging in meaningful activities together; disclosing personal, private thoughts and feelings and listening supportively to each other). Moderation analyses will examine whether these models may differentially apply depending on sex or role (patient or partner), or on the outcome targeted. Identifying differences in associations for these different subgroups will inform clinical application of the findings.

Use of technology in this study, specifically for EMA, will yield information regarding patients’ and partners’ smartphone access and their willingness to use the devices for research-based information and intervention delivery. Delivery of intervention content could occur using ecological momentary intervention or other types of real-time, mhealth methods.

Study limitations include a focus on patients with a subset of cancer diagnoses and the exclusion of patients either in the earliest stage of disease or facing death within 6 months. In addition, couples electing to participate may be unrepresentative of the larger group of patients with cancer and their spouses, as patients experiencing significant effects of disease or treatment might decline participation on that basis or for other reasons. Couples might also be hesitant to engage in the video-recorded conversation, or be reluctant to discuss topics related to cancer and its treatment, as these might result in emotional discomfort.

While the multi-modal nature of the assessments used in the study are a strength, as they provide multiple sources of information from both patient and partner, it is possible that some of the study procedures could be reactive and potentially affect responses to others. For example, engagement in the EMAs or the conversation could conceivably affect responses to other assessments by heightening perceptions or awareness of certain issues. We attempt to mitigate this in part by collecting the baseline self-report measures prior to the conversation and EMA. It is also possible that couples might discuss their responses to the EMA or other portions of the study, reducing the independence of their responses. We attempt to mitigate this by asking couples not to discuss their responses with each other. We believe this potential threat to internal validity is relatively low, and is offset by the benefits of collecting data in real time and in naturalistic settings.

## Dissemination Plans

Results will be published in scientific journals, presented at scientific conferences, and conveyed to a larger audience through infographics and social media outlets. We will also share findings with key stakeholders (oncology providers, patients and partners) to inform intervention planning and implementation.

## Ethics Statement

The studies involving human participants were reviewed and approved by the Arizona State University Institutional Review Board and Duke Health Institutional Review Board. The patients/participants provided their written informed consent to participate in this study. Reliance agreements were established to cover human subjects activities at the University of Washington and the Fred Hutchinson Cancer Research Center, with reliance on Arizona State University’s Institutional Review Board.

## Author Contributions

LP, SL, FK, JR, TS, KS, JBu, DB, JBr, NB, JG, VS, KW, and SZ obtained funding for the study, LP and SL as Multiple Principal Investigators. LP and SL were as Multiple Principal Investigators. LP, SL, FK, JR, TS, KS, JBu, DB, JBr, and NB contributed to study conception. LP, SL, FK, JR, TS, KS, JBu, DB, JBr, NB, JG, VS, MT, BB, MF, DW, and KL contributed to study design. SL wrote the first full draft of the manuscript. LP, NG, JR, MT, MF, DW, DB, BB, KR, and KL wrote sections of the manuscript. All authors contributed to manuscript revision, read, and approved the submitted version.

## Conflict of Interest

The authors declare that the research was conducted in the absence of any commercial or financial relationships that could be construed as a potential conflict of interest.

## Publisher’s Note

All claims expressed in this article are solely those of the authors and do not necessarily represent those of their affiliated organizations, or those of the publisher, the editors and the reviewers. Any product that may be evaluated in this article, or claim that may be made by its manufacturer, is not guaranteed or endorsed by the publisher.
